# Delusional Disorder over the Reproductive Life Span: The Potential Influence of Menopause on the Clinical Course

**DOI:** 10.1155/2015/979605

**Published:** 2015-10-27

**Authors:** Alexandre González-Rodríguez, Oriol Molina-Andreu, Rafael Penadés, Marina Garriga, Alexandre Pons, Rosa Catalán, Miguel Bernardo

**Affiliations:** ^1^Barcelona Clinic Schizophrenia Unit (BCSU), Neurosciences Institute, Hospital Clinic of Barcelona, University of Barcelona, 170 Villarroel Street, 08036 Barcelona, Spain; ^2^Department of Mental Health, Hospital Universitari Mútua de Terrassa, 5 Doctor Robert Street, 08221 Terrassa, Spain; ^3^Barcelona Clinic Schizophrenia Unit (BCSU), Neurosciences Institute, Hospital Clinic of Barcelona, Department of Psychiatry and Clinical Psychobiology, University of Barcelona, 170 Villarroel Street, 08036 Barcelona, Spain; ^4^Centro de Investigación Biomédica en Red en Salud Mental (CIBERSAM), Institut d'Investigacions Biomèdiques August Pi i Sunyer (IDIBAPS), 149 Rosselló Street, 08036 Barcelona, Spain; ^5^Barcelona Clinic Schizophrenia Unit (BCSU), Bipolar Disorders Unit, Neurosciences Institute, Hospital Clinic of Barcelona, University of Barcelona, 170 Villarroel Street, 08036 Barcelona, Spain

## Abstract

*Background and Objectives*. Recent evidence supports an association between estrogen levels and severity of psychopathology in schizophrenia women. Our main goal was to investigate whether delusional disorder (DD) women with premenopausal onset and those with postmenopausal onset differ in demographic and clinical features. *Methods*. Psychopathological symptoms were assessed in 80 DD women (DSM-IV-TR), at baseline and after six and 24 months. Scores in the PANSS, PSP for functionality, HRSD 17 items, C-SSRS for suicide, and the SUMD were considered outcome variables. For comparison purposes, *t*- and *χ* ^2^-tests were performed and nonparametric tests when necessary. Analysis of Covariance (ANCOVA) was conducted for multivariate comparisons. *Results*. 57 out of 80 DD women completed the study. When unadjusted, DD with premenopausal onset had a longer DUP, higher educational level, and a tendency toward higher rates of gynaecological disorders. Erotomanic type was most frequent in DD women premenopausal onset, and somatic and jealous types were most frequent in those with postmenopausal onset. After 24 months, DD women with premenopausal onset showed higher depressive symptoms and a tendency toward higher rates of psychotic relapses. *Conclusions*. Our results support that some aspects of psychopathology and insight may differ according to the onset of DD and the reproductive status.

## 1. Introduction

In recent years, an increasing scientific evidence highlighted an influence of estrogens on the psychopathology in women diagnosed with chronic psychosis [1, 2]. Most of the existing studies support an inverse correlation between estrogen levels and severity of psychopathology in these women [1, 3], and estradiol has been postulated to have specific antipsychotic-like effects on these populations.

For instance, Bergemann and colleagues investigated the effects of the menstrual cycle phase and estradiol levels on psychotic symptoms by using the Positive and Negative Syndrome Scale (PANSS) and the Brief Psychiatric Rating Scale (BPRS) [1]. The authors found a significant improvement in psychotic symptoms during the luteal phase of the menstrual cycle.

In this line of clinical and epidemiological findings, and based on animal models, two hypotheses have been proposed: “the estrogen protection hypothesis” and “the hypothesis of hypoestrogenism” [4, 5]. Specifically, the first hypothesis supports the notion that estrogens have a positive influence on mental well-being in women diagnosed with schizophrenia, and these women would be protected against the onset of the disorder between puberty and menopause [3, 5]. The second hypothesis proposed that schizophrenia women show a phenotype associated with a dysfunction of the estrogen and gonadal axis, irrespective of the use of antipsychotic medications.

When focusing on the estrogen protection hypothesis, a recent review proposed that women are more likely to develop psychotic disorders at a later age and show higher psychotic symptoms over the perimenopausal period [4]. As it was mentioned above, some clinical trials found new types of estrogen compounds to be an effective potential treatment for schizophrenia, in particular for those suffering from negative and cognitive symptoms [4, 5].

In particular, menopause has been defined as a medical condition with a permanent hypergonadotrophic hypogonadism and amenorrhea that determines a follicular depletion and a decline in estrogen serum levels [5]. This loss of estrogens would contribute to a high risk of onset of schizophrenia and deterioration of course in psychotic women. Furthermore, this depletion would also imply a high vulnerability for the exacerbation of psychotic symptoms in women previously diagnosed with schizophrenia [5].

Although increasing evidence regarding this issue in schizophrenia is provided, few studies have investigated differences in the psychopathology and clinical course of delusional disorder (DD) women with premenopausal onset and those with postmenopausal onset. Our team has recently published preliminary results focused on postmenopausal women, in a sample formed by 25 DD women, by describing some gynaecological and clinical endocrinological aspects in a descriptive design [2].

Thus, our main objective therefore was to investigate whether DD women with premenopausal onset and those with postmenopausal onset differ in sociodemographic characteristics, as it is a well-established fact that menopause interacts with other psychosocial risk factors and aging. Further, after comparisons in terms of sociodemographic characteristics, we aimed to search for differences in clinical features and psychopathology that means a comparison of the clinical course of the disorder between those women who had a background of menopause and other potential risk factors (e.g., educational level, employment status, and marital status) and those who had an onset prior to the menopause. We also aimed to compare longitudinally changes in psychopathology, rates of relapses, and rehospitalization rates between those who had an onset of DD prior to the menopause and those with a postmenopausal onset.

## 2. Methods

### 2.1. Patients and Study Design

We carried out a prospective observational study with a 24-month follow-up in a clinical group of 80 DD women, who attended for a first time our outpatient Schizophrenia Unit. We included all consecutive cases of DD women at the time they attended at psychiatric appointments. Our unit is of reference in a representative catchment area in Barcelona in the diagnosis, treatment, and research of schizophrenia and related disorders. Patients were evaluated at baseline and after six and 24 months.

Patients were included in the study if they met our inclusion criteria: (1) being aged >18 years, (2) being diagnosed as having a DD according to DSM-IV-TR criteria, and (3) being under antipsychotic treatment for more than six months. Female patients with a previous diagnosis of psychotic disorder, affective disorders or mental retardation, organic psychosis, or substance abuse were not invited to participate.

Age at onset of DD was determined by using the SCID-I interview that was assessed by at least two senior psychiatrists. The psychotic screening module for SCID-I has been widely used for coding psychotic symptoms that have been present at any point in the person's lifetime and can be used for clinical and research settings. Substance use disorders, general medical conditions, and mood disorders were excluded according to DSM-IV-TR criteria for DD. Further, onset of the disorder was obtained irrespective of the intensity of delusions.

The present study is a part of an ongoing study on schizophrenia and related disorders, which was approved by the Ethics Committee of the Hospital Clinic.

### 2.2. Measures and Psychopathological Assessment

Baseline sociodemographic and clinical variables were recorded, such as age, marital and employment status, educational level, age at onset of DD, age at first psychiatric appointment, DD type, and frequency of sexual delusional content. Relapse was defined as the occurrence or worsening of psychotic symptoms leading to hospitalization or to the need of higher rates for clinical appointments.

To investigate psychopathological symptoms, we used the following assessment tools at baseline and after six and 24 months: the Positive and Negative Syndrome Scale (PANSS) [6], which is a validated and translated scale for the assessment of positive, negative psychotic symptoms, the Personal and Social Performance Scale for functionality (PSP) [7], the 17-item Hamilton Rating Scale for Depression (HRSD-17) [8], the Columbia Suicide Severity Rating Scale (C-SSRS) [9] to identify lifetime and follow-up suicidal ideation and suicidal behaviour, and the first three items of the Scale to Assess Unawareness of Mental Disorder (SUMD) [10] Spanish version. Mean scores of the assessment scales at the six- and 24-month evaluation were considered outcome variables.

The sample was divided into two groups according the illness onset and the reproductive stage of the patients: group 1 was formed by DD women with premenopausal onset, and group 2 consisted of DD women with postmenopausal onset. The analysis was not intention to treat as we carried out a naturalistic and longitudinal study by comparing two subgroups of DD women in a pragmatic design. Patients were not excluded according to the adherence to protocol, so patients were grouped into two categories irrespective of the final results.

To classify DD women in premenopausal or postmenopausal groups, we account for the definitions from the International Menopause Society and the American Association of Clinical Endocrinologists, who have developed medical guidelines for clinical practice for diagnosis and treatment of menopausal women [11, 12]. According to these definitions, menopause was considered the permanent cessation of menstruation, defined by one year without menses, resulting from loss of ovarian activity. Postmenopause was the time period after menopause, and premenopause was the time period before this year without menstruations. DD women with a perimenopausal onset of the disease were not included in the study as the main aim of the study was to compare women with an onset disease prior to the menopause and those women with a postmenopausal onset of DD.

### 2.3. Statistical Analysis

All data were analyzed by using SPSS for Windows (Version 19.9; SPSS Inc., Chicago, Illinois, USA). Differences in sociodemographic and clinical characteristics and assessment scales between patient groups (DD women with premenopausal onset and postmenopausal onset) were tested by using *t*- and *χ* ^2^-tests for continuous or categorical variables and nonparametric tests when necessary. To investigate multivariate differences, Analysis of Covariance (ANCOVA) was applied. Mean scores of the assessment scales at six and 24 months served as dependent variables; patient's menopausal onset status as between-subject factor and duration of untreated psychosis (DUP), antipsychotic dosage in chlorpromazine equivalent doses, educational level, and baseline assessment scores were considered covariates.

Significance level was set at 0.05 (two-tailed).

## 3. Results

### 3.1. Description of the Total Sample

Eighty DD women were recruited, from those 25 (31.25%) had a premenopausal onset, and 55 (68.75%) had a postmenopausal onset. Seventy-four participants from the initial sample completed the 6-month assessment and 57 were evaluated after 24 months, being 28.75% of patients lost at follow-up.

At baseline, in the whole sample, 42.5% of DD women have been never married and 60% had no cohabiters when searching for social network. Near 70% of the total sample showed delusions of persecution, which was the DD type most commonly found, followed by erotomania (11.3%) and jealousy (7.5%). Other types of delusions were less frequent. Further, 45% of the sample reported having a history of gynaecological illness at lifetime.

Baseline demographic and clinical features are shown in Table 1.

### 3.2. Baseline Comparisons of Sociodemographic, Clinical Features, and Psychopathology between Groups

DD women with premenopausal onset showed a longer DUP (*p* = 0.028) and had higher educational level (*p* = 0.026) compared to female DD with postmenopausal onset. No statistically significant differences were found between both groups in terms of other sociodemographic variables. Erotomanic type was more frequent in DD women with premenopausal onset, whereas jealous and somatic contents were more common in women with postmenopausal onset (*p* = 0.003). When analyzing the presence of sexual content in all types of delusions, women with premenopausal onset had higher rates of sexual delusional content compared to those with postmenopausal onset (*p* = 0.006).

At baseline, DD women with postmenopausal onset showed lower scores in the SUMD total (*p* = 0.023) than women with premenopausal onset. There were no significant differences in psychotic and depressive symptoms, neither in functionality and lifetime suicidal ideation and behaviour nor in intensity of lifetime suicidal ideation.

Mean differences in sociodemographic and clinical variables between groups are presented in Table 1.

### 3.3. Comparisons of Clinical and Psychopathological Symptoms at Follow-Up between Groups

After six months, no statistically significant differences were found between both groups in psychopathological symptoms, even when adjusted by confounding factors.

Furthermore, after 24 months, when uncorrected for potential confounders, no significant differences were found between both groups in psychotic symptoms, except for a tendency toward higher negative symptoms in those women with premenopausal onset (*p* = 0.057). On the other hand, DD women with premenopausal onset had also higher depressive symptoms compared to the other group (*p* = 0.008).

At the end of the follow-up period, despite being not statistically significant, DD women with premenopausal onset had higher rates of psychotic relapses (70% versus 54%) (e.g., at least one relapse) than women with postmenopausal onset.

After controlling for potential confounders, postmenopausal DD women showed a tendency toward higher scores in item 3 of the SUMD scale (e.g., awareness of social consequences) and had lower scores in depressive symptoms (*p* = 0.021) when compared to DD women with premenopausal onset. Differences in negative symptoms did not withstand significantly after adjustment.

**Figure 1:**
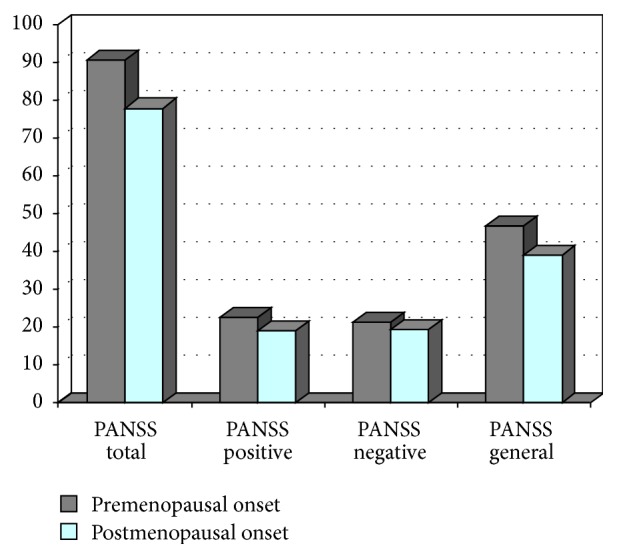
Assessment of psychotic symptoms after 24 months by groups.

Mean scores of psychotic and depressive symptoms and insight scores at the 24-month assessment can be found in Table 2.  Figure 1 also shows the multivariate comparisons of the PANSS between women with premenopausal onset and those women with postmenopausal onset.

## 4. Discussion

To date, the vast majority of gender specific literature has been focused on the study of gender differences in the psychopathology and clinical course of DD patients. The most recent study regarding this issue could not confirm that male and female DD patients differ in age at onset, age at first psychiatric consultation, or suicidal ideation and behaviour, even after controlling for potential confounders [13]. However, to the best of our knowledge, no studies have investigated differences in the psychopathology of DD women attending to their reproductive status at the onset of the illness.

Thus, we conducted a prospective observational study with a 24-month follow-up period in DD women for investigating whether DD women with premenopausal onset and those with postmenopausal onset differ in sociodemographic and clinical features and clinical course of the disorder. In this field, the only work focused on postmenopausal DD women was carried out by our team, as a first phase of an ongoing study [2]. This preliminary study evaluated gynecological variables in postmenopausal DD patients, and no correlations between age at menopause and clinical variables could be found.

In our study, surprisingly, DD women who presented a premenopausal onset of the disorder had a longer DUP and higher educational level compared to female DD patients with postmenopausal onset, suggesting that psychopathological and clinical course comparisons between groups should be controlled by these potential confounding variables. With regard to the content of delusions, erotomanic type was more frequent in women with premenopausal onset, and jealous and somatic themes were more frequent in those female DD patients with postmenopausal onset. The most representative study investigating gender differences in clinical symptoms in DD patients found no differences in DD type between men and women [14]. However, we hypothesized that these differences found in delusional content may be present when taken into account the reproductive status in women diagnosed with DD. In line with our findings, several authors have emphasized that psychotic phenomena, such as delusional content, may be related to sociodemographic and cultural variables and gender. For instance, delusional content can be related to social isolation in later life, an association not surprisingly found in women [15, 16]. In agreement with these findings, it has been reported that tactile and visual nonprominent hallucinatory phenomena may be related to late-onset psychosis, strongly associated with somatic delusional types, which were found to be more frequent in DD women with postmenopausal onset in our sample [17]. Further, some studies have highlighted that these well-established gender differences in the content of delusions can be interpreted by biological sex factors and cross-cultural factors, and not only by the age at onset of disease. In summary, different types of delusions between DD women with premenopausal onset and those with postmenopausal onset should be interpreted by the complex interactive contribution of biological, psychosocial, and cultural factors involved in the menopausal period, which is one of the most important social and biological changes in life span [16]. Further, the interface between aging and menopause should be considered, as aging can interact with the reproductive status, and menopause, defined as a loss of estrogen function, may have an influence on the process of aging [5]. For instance, estrogen withdrawal in the menopause has been associated with increased vulnerability of cerebrovascular disease, which several authors consider a risk factor for late-onset psychosis [17].

We found no baseline differences between both groups in terms of psychotic and depressive symptoms, functionality, and suicidality. To date, no studies have compared these clinical features in women with premenopausal and postmenopausal onset. de Portugal and colleagues [14] found that DD men showed a worse functioning and a more severe illness compared to women, but the potential effects of menopause on the clinical course in DD women were not specifically investigated.

Psychopathological symptoms, insight, rates for relapses, and admission rates at follow-up between both groups were also compared. After six months, these clinical variables did not seem to differ between DD women with premenopausal or postmenopausal onset. However, at the end of the study, when unadjusted for confounding factors, DD women with premenopausal onset showed a tendency toward higher negative symptoms compared to the other group. These findings are in agreement with previous studies that highlighted the potential vulnerability of women for an exacerbation of psychotic symptoms or a worsening of psychotic disorders in the menopause [4, 5, 18] and consistent with the fact that the loss of the protective effect of estrogens may be followed by a deterioration of course in women suffering from psychosis diagnosed in the reproductive period. In summary, this loss of protection after menopause may explain the presence of higher rates of relapses and higher depressive symptoms in women diagnosed with psychosis during the reproductive life span (e.g., DD women with premenopausal onset). On the other hand, lower depressive symptoms in DD women with postmenopausal onset compared to women with premenopausal onset can be explained by the fact that we did not include DD women in the perimenopausal period. In brief, the perimenopausal transition seems to be characterized by significantly higher intensity of depressive symptoms when compared to life span after menopause, and this fact may explain why DD women with postmenopausal onset did not achieve higher severity of depressive symptoms compared to postmenopausal women with an onset in the premenopausal period. In our study, no statistically significant differences in terms of positive psychotic symptoms were found between both groups. This finding is also in line with several studies that found patients who develop psychosis in later life to have similar intensity of positive symptoms compared with patients with an earlier onset of disease [19, 20].

Moreover, in our sample, DD women with premenopausal onset showed higher rates of relapses and higher depressive symptoms at the end of the study period compared to DD women with postmenopausal onset. This fact is also in line with the studies aforementioned [4, 5, 18]. Women with premenopausal onset showed a worse global insight at the time of the study inclusion that could have mediated the recovery or worsening of psychotic symptoms, specifically in negative symptoms. Gumley and coworkers [21] explored the relationship between DUP, insight, and psychotic recovery in a prospective sample of first-episode of psychosis. The authors found that recovery in negative symptoms was predicted by both DUP and insight, which is in line with our findings. However, these findings should be confirmed in larger samples comparing women diagnosed with schizophrenia with females individuals suffering from DD.

In a further step, we considered DUP, severity of baseline psychopathological symptoms, antipsychotic doses (i.e., chlorpromazine equivalent dosage), and educational level as potential confounders, so they were included as covariates in multivariate analyses. After controlling for these factors, postmenopausal DD women showed a tendency toward worse insight in terms of awareness of social consequences of the disorder compared to women with premenopausal onset, suggesting that a later onset of the disease would have an influence on insight. These results in our study are supported by a recent review [22] proposing trajectories of insight in schizophrenia across the life span. The authors concluded that insight may be worse at the first-episode of psychosis, shows a moderate improvement in midlife, and declines in the elderly. Further support for the influence of menopause in an indirect consideration (i.e., aging) comes from a recent study in late-life schizophrenia [23], which suggests that predictors of insight in schizophrenia differ across the life span, probably according to age-related changes in neurocognitive performance.

*Strengths and Limitations.* In our point of view, this study provides unique evidence in the field of delusional disorders, as to best of our knowledge; the vast majority of studies have been focused on the investigation of gender differences without taken into account the potential influence of menopause on the psychopathology or the clinical course.

However, in our study several limitations should be mentioned: (1) the first limitation would be the small sample size that could have limited the statistical power and the possibility of performing more complex statistical multivariate analysis and (2) the second limitation would be the lack of blood determinations of estrogen levels in these DD women; as this was not the aim of our study. Furthermore, perimenopause was not considered index episode in our study.

## 5. Conclusions

In summary, it is apparent that erotomanic, jealous, and somatic delusional contents may vary across the reproductive life in DD women.

DD women who presented a premenopausal onset attended later to our outpatient service and presented higher depressive symptoms compared to women with postmenopausal onset, suggesting a worse insight at the study entry and a worsening of symptoms after the menopause in women with premenopausal onset. Further, this group of DD women showed a tendency toward higher negative symptoms that may be mediated by the DUP and insight scores aforementioned. Larger studies should confirm these findings and propose a global model to better explain our results.

DD women with postmenopausal onset had a tendency toward a worse insight in social consequences after 24 months, suggesting that aging may have an influence on the course of insight in the direction of worse insight in the elderly.

## Figures and Tables

**Table 1:** Baseline sociodemographic and clinical features and psychopathology symptoms by clinical groups (*N* = 80).

Variable	Total sample *N* = 80	DD women premenopausal onset *n* = 25	DD women postmenopausal onset *n* = 55	*p* value
Sociodemographic variables				
DUP, mean years (SD)	5.40 (9.5)	8.84 (13.9)	3.84 (6.01)	*t* = 2.243, df = 80, *p* = 0.028^*∗*^
Marital status, *n* (%)				*χ* ^2^ = 1.482, df = 2, *p* = 0.477
Never married	34 (42.5)	13 (52)	21 (38.2)	
Married/partner	17 (21.3)	5 (20)	12 (21.8)	
Divorced/separated/widowed	29 (36.3)	7 (28)	22 (40)	
Social network (cohabiters), *n* (%)				*χ* ^2^ = 1.273, df = 3, *p* = 0.735
0	48 (60)	15 (60)	33 (60)	
1	23 (28.8)	6 (24)	17 (30.9)	
2	6 (7.5)	3 (12)	3 (5.5)	
3 or >	3 (3.8)	1 (4)	2 (3.6)	
Educational level (years), *n* (%)				*χ* ^2^ = 9.225, df = 3, *p* = 0.026^*∗*^
<8	10 (12.5)	1 (4)	10 (12.5)	
8-9	29 (36.3)	7 (28)	29 (36.3)	
10-11	22 (27.5)	6 (24)	22 (27.5)	
12 or >	19 (23.8)	11 (44)	19 (23.8)	
Employment status, *n* (%)				*χ* ^2^ = 6.303, df = 3, *p* = 0.098
Unemployed	6 (7.5)	2 (8)	4 (7.3)	
Employed	24 (30)	8 (32)	16 (29.1)	
Economic benefit	20 (25)	10 (40)	10 (18.2)	
Pensioner	30 (37.5)	5 (20)	25 (45.5)	

Clinical features				
DD type, *n* (%)				*χ* ^2^ = 6.315, df = 4, *p* = 0.003^*∗*^
Persecutory	55 (68.8)	14 (56)	41 (74.5)	
Erotomanic	9 (11.3)	8 (32)	1 (1.8)	
Jealous	6 (7.5)	1 (4)	5 (9.1)	
Somatic	3 (3.8)	1 (4)	2 (3.6)	
Grandiose	7 (8.8)	1 (4)	6 (10.9)	
Sexual delusional content, *n* (%)	19 (23.8)	11 (44)	8 (14.5)	FET = 0.009^*∗*^
Lifetime suicidal behaviour, *n* (%)	14 (17.5)	5 (20)	9 (16.4)	*χ* ^2^ = 0.157, df = 1, *p* = 0.755
Gynaecological disorders, *n* (%)	36 (45)	15 (60)	21 (38.2)	FET = 0.058

Psychopathological symptoms, mean (SD)				
PANSS total	88.04 (14.44)	86.48 (21.91)	88.75 (15.16)	*t* = −0.536, df = 78, *p* = 0.642
PANSS positive subscale	24.77 (6.69)	24.68 (7.53)	24.82 (6.34)	*t* = −0.085, df = 78, *p* = 0.932
PANSS negative subscale	17.86 (5.18)	18.40 (6.92)	17.62 (4.21)	*t* = 0.624, df = 78, *p* = 0.605
PANSS general subscale	45.4 (8.87)	43.40 (10.54)	46.31 (7.94)	*t* = −1.367, df =78, *p* = 0.175
PSP	48.74 (13.37)	51.36 (16.2)	47.55 (11.87)	*t* = 1.186, df = 78, *p* = 0.724
HRSD-17	9.74 (5.92)	10.72 (6.52)	9.29 (5.63)	*t* = 1.001, df =78; *p* = 0.320
SUMD total	14.25 (1.45)	14.72 (1.02)	14.04 (1.65)	*t* = 2.329, df = 66.9, *p* = 0.023^*∗*^
C-SSRS, suicidal ideation intensity	3.39 (7.01)	3.12 (6.53)	3.51 (7.38)	*t* = −1.208, df = 71, *p* = 0.172

^*∗*^ *p* < 0.05.

C-SSRS: Columbia Suicide Severity Rating Scale; DD: delusional disorder; df: degrees of freedom; DUP: duration of untreated psychosis; FET: Fisher's exact test; HRSD: Hamilton Rating Scale for Depression; *p*: *p* value; PANSS: Positive and Negative Syndrome Scale; PSP: Personal and Social Performance Scale; SD: standard deviation; SUMD: Scale for Unawareness of Mental Disorder; *t*: Student *t*-test; *χ* ^2^: Chi-square test.

**Table 2:** Psychopathological scores after 24 months adjusted for covariates and multivariate comparisons by groups.

Dependent variables, mean scores (SD)	Premenopausal DD women	Postmenopausal DD women	Two-way ANCOVA
PANSS total	90.51 (5.28)	77.90 (2.99)	*F*(1,41) = 0.089, *p* = 0.767
PANSS positive subscale	22.48 (1.57)	18.99 (0.88)	*F*(1,41) = 2.614, *p* = 0.114
PANSS negative subscale	21.43 (1.99)	19.35 (1.12)	*F*(1,41) = 9.192, *p* = 0.567
PANSS general subscale	46.73 (2.45)	39.09 (1.38)	*F*(1,41) = 3.971, *p* = 0.757
SUMD total	9.88 (1.15)	10.78 (0.43)	*F*(1,41) = 4.590, *p* = 0.291
SUMD item 1	3.44 (0.46)	3.93 (0.18)	*F*(1,41) = 0.054, *p* = 0.782
SUMD item 2	3.71 (0.50)	3.43 (0.18)	*F*(1,41) = 0.396, *p* = 0.467
SUMD item 3	2.73 (0.49)	3.38 (0.18)	*F*(1,41) = 2.236, *p* = 0.082
HRSD-17	8.83 (1.09)	3.91 (0.62)	*F*(1,41) = 2.708, *p* = 0.021^*∗*^

^*∗*^ *p* < 0.05.

DD: delusional disorder; HRSD: Hamilton Rating Scale for Depression, PANSS: Positive and Negative Syndrome Scale; PSP: Personal and Social Performance Scale; SD: standard deviation; SUMD: Scale to Assess Unawareness of Mental Disorder.
